# Case report: Improvement of gait with adaptive deep brain stimulation in a patient with Parkinson’s disease

**DOI:** 10.3389/fbioe.2024.1428189

**Published:** 2024-09-11

**Authors:** Ioannis U. Isaias, Laura Caffi, Linda Borellini, Antonella M. Ampollini, Marco Locatelli, Gianni Pezzoli, Alberto Mazzoni, Chiara Palmisano

**Affiliations:** ^1^ Parkinson Institute of Milan, ASST G.Pini-CTO, Milano, Italy; ^2^ University Hospital of Würzburg and Julius Maximilian University of Würzburg, Würzburg, Germany; ^3^ The BioRobotics Institute, Sant’Anna School of Advanced Studies, Pisa, Italy; ^4^ Foundation IRCCS Ca’ Granda Ospedale Maggiore Policlinico, Milano, Italy; ^5^ Department of Excellence in Robotics and AI, Sant’Anna School of Advanced Studies, Pisa, Italy

**Keywords:** adaptive deep brain stimulation, Parkinson’s disease, gait, subthalamic nucleus, local field potentials

## Abstract

Gait disturbance is a common and severe symptom of Parkinson’s disease that severely impairs quality of life. Current treatments provide only partial benefits with wide variability in outcomes. Also, deep brain stimulation of the subthalamic nucleus (STN-DBS), a mainstay treatment for bradykinetic-rigid symptoms and parkinsonian tremor, is poorly effective on gait. We applied a novel DBS paradigm, adjusting the current amplitude linearly with respect to subthalamic beta power (adaptive DBS), in one parkinsonian patient with gait impairment and chronically stimulated with conventional DBS. We studied the kinematics of gait and gait initiation (anticipatory postural adjustments) as well as subthalamic beta oscillations with both conventional and adaptive DBS. With adaptive DBS, the patient showed a consistent and long-lasting improvement in walking while retaining benefits on other disease-related symptoms. We suggest that adaptive DBS can benefit gait in Parkinson’s disease possibly by avoiding overstimulation and dysfunctional entrainment of the supraspinal locomotor network.

## 1 Introduction

Deep brain stimulation (DBS) of the subthalamic nucleus (STN) is a mainstay treatment for Parkinson’s disease (PD). However, poorly manageable axial symptoms such as gait derangements and subsequent falls may emerge along with disease progression, adding to the global clinical burden and causing significant disability (Pozzi and Isaias, 2022; Pozzi et al., 2022). On the whole, the response of gait impairment to DBS is often unsatisfactory and, in some instances, treatment-induced worsening is possible, as DBS can directly interfere with the physiological, integrated functioning of the cortical, subcortical and spinal components of the locomotor network (Palmisano et al., 2020b). In particular, the delivery of DBS with constant stimulation parameters (i.e., conventional DBS, cDBS) may alter the dynamic synchronization between cortical areas involved in motor control, the basal ganglia and the mesencephalic locomotor regions, thus directly impairing gait adaptation to contextual needs (Chen et al., 2006; St George et al., 2010; Reich et al., 2016; Pozzi et al., 2019). To increase therapeutic effect, novel adaptive DBS (aDBS) systems are being developed (Arlotti et al., 2019; Bocci et al., 2021; Guidetti et al., 2021; Thenaisie et al., 2021). These devices operate by adapting the stimulation parameters in real-time in response to a neural input signal that can represent symptoms, motor activity, or other behavioral features. In currently available devices (AlphaDBS, Newronika S.p.A. and Percept™, Medtronic Inc.) the biomarker implemented for aDBS is the oscillatory beta activity (13–30 Hz) of the local field potentials (LFP) recorded at the implanted site. This biomarker was chosen because beta activity correlates with bradykinetic-rigid parkinsonian symptoms and it is modulated by dopaminergic treatment and DBS (Yin et al., 2021). This aDBS mode has been shown to provide better control of motor symptoms in the short term (lower UPDRS-III score and less dyskinesia) accompanied by less energy delivered than cDBS (Little et al., 2013; Rosa et al., 2017; Bocci et al., 2021). To date, there are no studies showing sustained clinical efficacy, especially on gait, of chronic treatment with aDBS. We now describe the clinical, neurophysiological and kinematic data of a patient with PD and bilateral STN-DBS who experience significant improvement in gait when changing from cDBS to aDBS mode.

## 2 Case description

We report the case of a male patient in his seventies with PD for 27 years and cDBS (Activa PC, 3389 leads, Medtronic Inc.) for 10 years, who started aDBS upon receiving the AlphaDBS neurostimulator at the second battery replacement. The motor symptoms of PD started in his mid-forties with rigidity and motor impairment in the right arm. A diagnosis of PD was made the same year and confirmed with SPECT with FP-CIT. The patient started treatment for about 3 years with pramipexole and rasagiline, then, after 5 years also with levodopa/carbidopa, with remarkable benefit. In his late-fifties peak-dose dyskinesias appeared, even at low doses of levodopa/carbidopa, worsening in severity and duration over time, and resulting in the patient being treated with STN-cDBS+ in his mid-sixties (i.e., dopaminergic medication was always continued, +). The daily levodopa equivalent dose (Jost et al., 2023) was reduced by 53% after surgery. Over time, the patient has always shown consistent and stable benefits from STN-cDBS+. About 4 years after the start of cDBS+, the patient began to complain of some difficulties in walking (UPDRS item 23 score: >1), with the appearance of freezing of gait and festination. Several stimulation programs were tried, and low frequencies (i.e., 70 Hz) (Pozzi et al., 2022) were chosen as improving gait problems to the most, even though insufficiently, with still remarkable benefit on bradykinetic-rigid signs. The parameters for chronic cDBS+ were: right STN, C+0-, 70 Hz, 60 μs, 3.5 mA and left STN, C+2-, 70 Hz, 60 μs, 4 mA. After receiving the AlphaDBS device, the same parameters were adopted during aDBS+ with current delivery ranging from 3.0 to 4.0 mA for the right STN and between 3.5 and 4.5 mA for the left STN, in order to maintain comparable total energy delivered (TEED). Pharmacological treatment (levodopa/carbidopa 100/50 mg TID, pramipexole 1.05 mg QD, and rasagiline 1 mg QD) was unchanged between cDBS+ and aDBS+.

## 3 Materials and methods

The study was approved by Milano Area 2 Ethics Committee (approval: 165-2020 and 93-2023bis) and conformed to the declaration of Helsinki. The patient gave written consent prior to participation in the study and for publication of the data.

### 3.1 Programming paradigm of adaptive DBS with the AlphaDBS device

In aDBS mode, the AlphaDBS device (Arlotti et al., 2021) applies a linear algorithm that changes the stimulation current every minute based on the average LFP amplitude calculated in a patient-specific beta frequency range (BFRA) and normalized for the total amplitude in the 5–34 Hz range. Specifically, the averaging procedure consists of an exponential moving average with a time constant of 50 s over the beta samples calculated with 1-s resolution. The stimulation amplitude is adjusted within a pre-defined clinically effective range (i.e., Amin and Amax), while the stimulation frequency and pulse width remain fixed.

**FIGURE 1:**
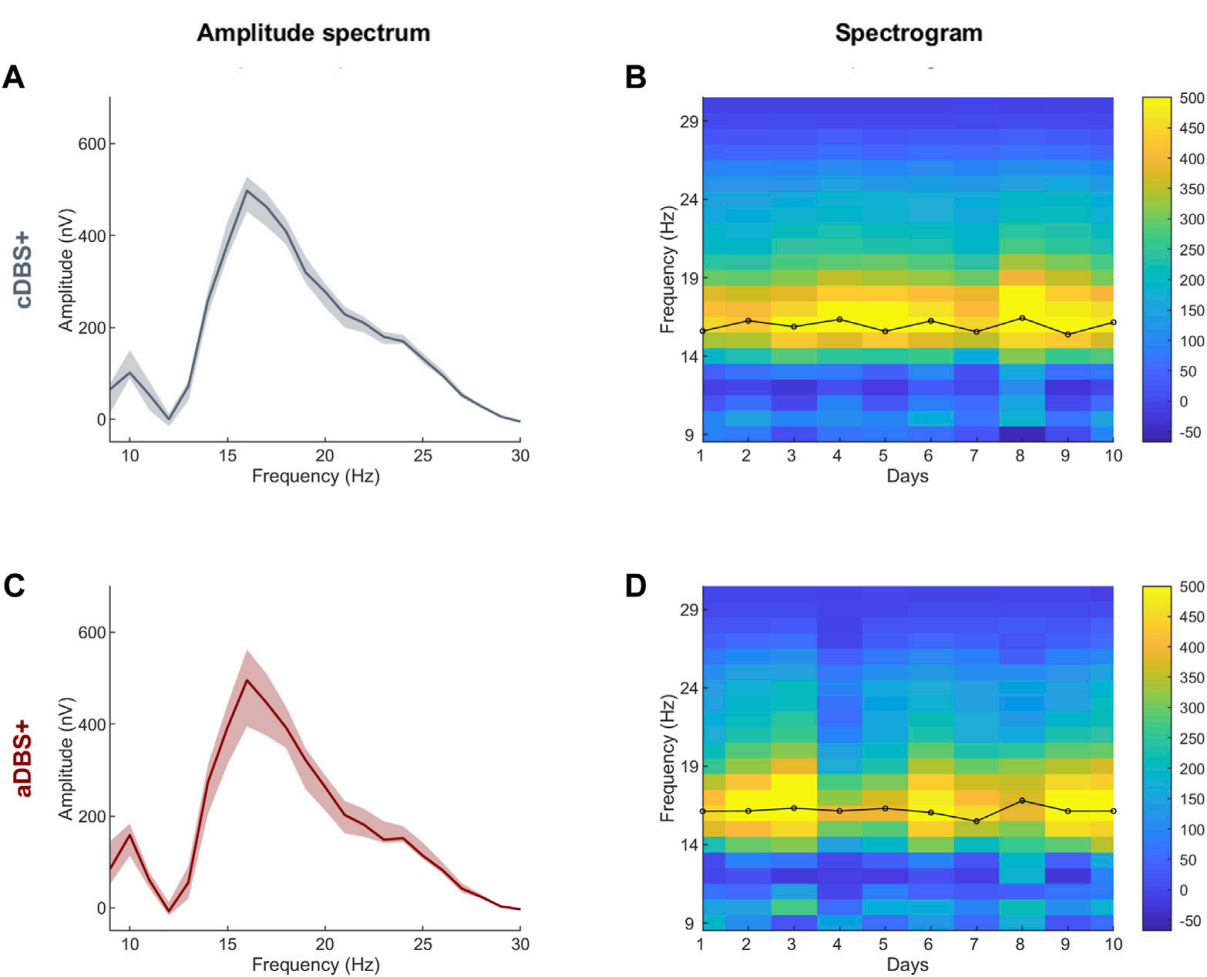
STN-LFP daily spectra in cDBS+ and aDBS+ **(A)** Median (solid line) of the daily mean amplitude spectra during the awake time of the 10 days in cDBS+. The daily mean spectra are cleaned from 1/*f^n^* noise. The dashed area is bound by the first and third quartiles of the daily mean amplitude spectra. **(B)** Spectrogram of daily mean amplitude spectra cleaned from 1/*f^n^* noise during awake time. Black line on top of the spectrogram represents the time course of the central frequency of the beta peak identified in each daily mean amplitude spectrum during the awake time. **(C)** Same as **(A)** during aDBS+. **(D)** Same as **(B)** during aDBS+. Abbreviations: a, adaptive; c, conventional; DBS, deep brain stimulation; DBS+, with dopaminergic medication; LFP, local field potentials; STN, subthalamic nucleus.

**FIGURE 2:**
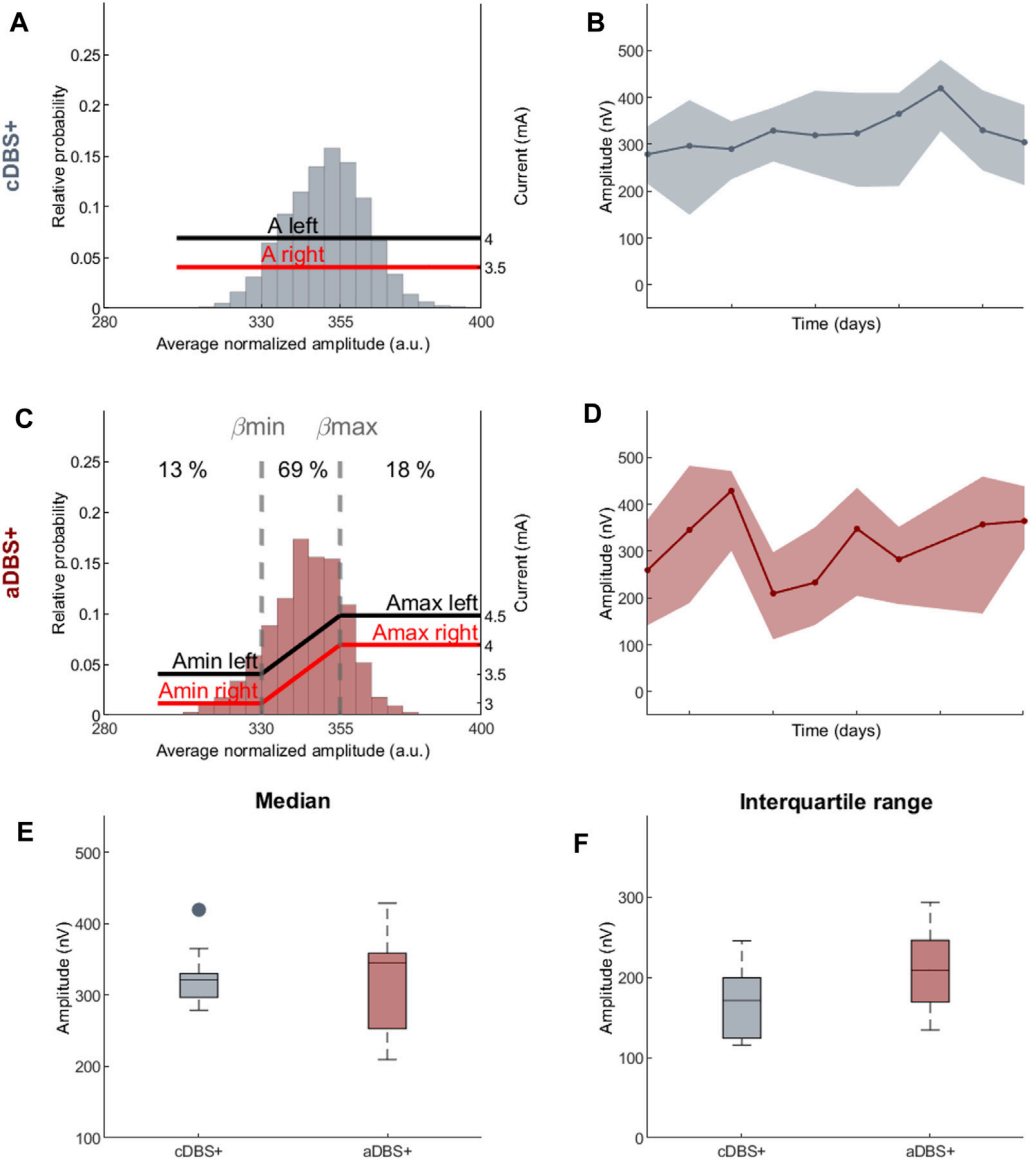
Programming paradigms and STN-LFP recordings **(A)** Parameter settings and probability distribution of the biomarker (amplitude of the right STN in the 12–19 Hz range normalized over the total amplitude in the 5–34 Hz range) acquired during awake time for 10 days in cDBS+ mode. In cDBS+ mode, independently from the value read minute by minute, the current remains fixed at a clinically defined level (red and black lines, respectively, for the right and left STN). **(B)** Evolution of the daily median BFRA during the awake period (solid line) in cDBS+. The shadowed area is bound by the daily first and third quartile of the BFRA. **(C)** Same as **(A)** for 10 days in aDBS+ condition. Vertical dotted lines represent the biomarker limits for current adjustment (βmin and βmax). Red and black solid lines represent the stimulation current at a specific reading. Numbers on top show the time percentage of STN-LFP amplitude being less than βmin, between βmin and βmax and above βmax in the considered 10 days period. **(D)** Same as **(B)** for aDBS+ condition. **(E)** Boxplot of the BFRA daily median during the awake period in cDBS+ and aDBS+ **(F)** Same as **(E)** for the interquartile range of the BFRA. Abbreviations: a, adaptive; A, pre-defined, clinically-effective amplitude; β, average normalized beta amplitude; c, conventional; BFRA, patient-specific beta frequency range amplitude; DBS, deep brain stimulation, DBS+, with dopaminergic medication; LFP, local field potentials; STN, subthalamic nucleus.

In this patient, the right recording contact pair one to two was chosen to identify the biomarker for aDBS as the one showing the most prominent and stable beta peak (on data collected on 1 day of cDBS+). The frequency range monitored (12–19 Hz) was ±3.5 Hz centered on the beta frequency peak (16 Hz). Very important for proper aDBS programming is that the frequency peak always remained within the range set for this patient. In some cases, multiple peaks were detected within the beta range, however, the one at 16 Hz was always the most prominent and stable (Figure 1). The beta amplitude distribution in this frequency range allowed the identification of beta amplitude limits (βmin and βmax) by which the stimulation current was to be delivered in aDBS condition (Figure 2C). In terms of programming options, these limits allow to define the amount of time the patient is stimulated at Amin or Amax.

The two beta limits (βmin and βmax) were set based on a cumulative distribution of 24 h recording in cDBS+ to properly capture beta power fluctuations. We allowed amplitude modulation with intermediate current values between Amin and Amax for about 70% of the total time (Figure 2C). As a result, during the 10 days of aDBS+, the patient spent a total of 13% of the time with Amin and 18% with Amax. Importantly, the time spent in Amin and Amax was generally not consecutive: for only 8% of the time of the 10 days with aDBS+ did the current varied less than 2 mA in windows of at least 30 consecutive minutes (Supplementary Figure S1).

DBS programming in cDBS was performed as standard of care (Değirmenci, 2024). For aDBS, the two stimulation current limits were clinically defined as the amperage (Amin) with 40%–50% benefit in meds-off state (i.e., titrating up the stimulation current in the morning after overnight suspension of all dopaminergic drugs) and the maximum amperage (Amax) in the absence of side effects in meds-on condition (titrating up the stimulation current at 60 min after 100/50 mg levodopa/carbidopa intake).

### 3.2 Movement analysis

Clinical evaluation and gait analysis were performed after 1 month of chronic and stable aDBS+. The patient was then reprogrammed with the original cDBS+ settings for 10 days and a second clinical assessment and gait analysis were performed (see Results). Both clinical and gait assessments were performed in the morning starting 90 min after the intake of morning medications, with chronic aDBS+ or cDBS+ (i.e., best medical treatment). The evaluating physician was blind to the treatment of the patient.

In each stimulation mode, we recorded three posturography recordings (30 s), each followed by a gait initiation trial. We also recorded three unperturbed barefoot linear walking trials (on a 10 m walkway). The patient was allowed to rest between trials.

The patient was instructed not to make any voluntary movements (e.g., turning head, talking, lifting arms, etc.) that would have invalidated posturography and gait recordings. The validity of the acquisitions was checked with video recordings synchronized with the kinematic data.

Motor performance was monitored with a six-camera optoelectronic system (SMART-DX, BTS) and two dynamometric force plates (P-6000, BTS). Four markers were placed bilaterally on the two feet on the main anatomical landmarks (heels, outer ankle bones, fifth metatarsi and hallux tips) to allow the identification of gait cycle events (heel-off and toe-off) and the computation of gait spatiotemporal parameters (Palmisano et al., 2022b). Three additional markers were attached to the pelvis (on the anterior superior iliac spines and the middle point between the posterior superior iliac spines) to allow the estimation of the center of mass (CoM) at gait initiation (Palmisano et al., 2022b).

For the gait analysis, kinematic variables were averaged over all available strides. One gait initiation trial in cDBS+ condition was excluded from the analysis due to a technical failure which prevented data recording. Anticipatory postural adjustments (APAs) preceding the first step at gait initiation were subdivided into the imbalance and the unloading phase (Palmisano et al., 2020b; Palmisano et al., 2020a; Palmisano et al., 2022a). The length of the center of pressure (CoP) pathway and the duration of the two phases were computed as indicators of the quality of motor programming and execution. The length and velocity of the first step were calculated from foot marker traces, along with the CoM velocity at the toe-off of the stance foot, as measures of the effectiveness of gait initiation. During standing, we computed the CoP trajectory length, medio-lateral and anterior-posterior range of CoP oscillation, and the confidence ellipse containing the 95% of the CoP samples (Farinelli et al., 2020), as measures of balance control (Farinelli et al., 2020). For walking and gait initiation, outcome measures were averaged across trials and standard deviation computed as an estimation of gait variability, while standing variables were computed over the trial duration. Considering the low number of trials for the posturography and gait initiation assessments, formal statistical comparisons between aDBS+ and cDSB+ were performed only for stride parameters during walking (Table 1). The effect of treatment on gait cycle parameters was evaluated with a parametric (t-test) or a non-parametric test (Mann-Whitney U test) according to data distribution, investigated with the Anderson-Darling test. The statistical significance was set at 0.05.

**TABLE 1:** Kinematic measurements. Kinematic measurements for walking, posturography, and gait initiation in aDBS+ and cDBS+ conditions. For each stimulation condition, values are reported as mean (standard deviation) across available trials. Three walking and gait initiation trials were considered for each stimulation condition for the analysis. One gait initiation trial in cDBS+ was excluded due to a technical failure. Posturography measures were assessed based on a 30 s trial for each stimulation condition. The confidence ellipse was drawn as the ellipse containing the 95% of the points of the CoP tracks, and axes a and b were computed as its major and minor axes, respectively. CoM was estimated as the barycenter of the triangle described by the three markers placed on the pelvis. APAs were subdivided into imbalance and unloading phases, according to reference points identified on the CoP track (Figure 3). For further details on posturography and gait initiation elaboration please refer to (Palmisano et al., 2020b; Palmisano et al., 2020a; Palmisano et al., 2022a). *p < 0.05, t-test or Mann-Whitney U test as appropriate. Statistical comparisons were performed only between walking measurements. Abbreviations: a, adaptive; APAs, Anticipatory Postural Adjustments; c, conventional; CoM, centre of mass; CoP, centre of pressure; DBS, deep brain stimulation; DBS+, with dopaminergic medication.

	aDBS+	cDBS+
WALKING		
Stride duration (s)	1.42 (0.10) *	1.12 (0.25) *
Stride length (m)	0.79 (0.06) *	0.41 (0.11) *
Stride average velocity (m/s)	0.56 (0.04) *	0.38 (0.11) *
Stride maximal velocity (m/s)	1.97 (0.13) *	1.53 (0.30) *
Stance duration (%gait cycle)	68.22 (2.21)	70.01 (4.61)
POSTUROGRAPHY		
Base of support (cm^2^)	606.73	614.77
CoP length (mm)	537.72	540.51
Medio-lateral CoP range (mm)	30.81	36.68
Anterior-posterior CoP range (mm)	46.24	41.37
Ellipse area (mm^2^)	711.56	859.34
Axis a ellipse (mm)	18.74	20.55
Axis b ellipse (mm)	12.08	13.31
GAIT INITIATION		
Imbalance duration (s)	0.44 (0.16)	0.33 (0.18)
Imbalance CoP length (mm)	39.75 (22.35)	19.93 (8.32)
Unloading duration (s)	0.50 (0.36)	1.13 (0.15)
Unloading CoP length (mm)	94.78 (29.08)	117.85 (16.43)
First step length (m)	0.32 (0.08)	0.23 (0.04)
First step average velocity (m/s)	0.47 (0.13)	0.24 (0.03)
Toe-off stance CoM velocity (m/s)	0.45 (0.07)	0.26 (0.08)

### 3.3 Spectral analysis

During the last 10 days of aDBS+ and the 10 days of cDBS+, we recorded during home monitoring bilateral stimulation amplitude, unilateral (right) average STN-LFP spectra every 10 min and unilateral (right) amplitude in the patient-specific beta frequency range every minute. We then analyzed the data recorded during the awake period (9a.m.-10p.m., based on the patient’s daily routine).

To investigate relevant spectral peaks, we cleaned the amplitude spectra from 1/*f^n^* noise as follows. For each day, the average spectra showed an aperiodic component superimposed on the oscillatory peaks. Consequently, the daily average spectra were decomposed in the aperiodic and periodic components, modeled respectively as exponential functions in a semi-logarithmic amplitude-space with characteristic offset, slope and bend, and gaussian functions with characteristic central frequency, amplitude and width (Donoghue et al., 2020). The quality of the decomposition was visually inspected and days presenting residual spectral artifacts after subtraction of the aperiodic component were removed by the analysis. The presence and stability of the gaussian peaks were inspected across days and conditions. For each day, the daily periodic component was then subtracted from each 10-min amplitude spectrum. Within each day we calculated the median and interquartile range of the BFRA (STN-LFP amplitude in the patient-specific beta frequency range) across all 10-min amplitude spectrum acquired along the awake period. The effect of treatment (cDBS+ and aDBS+) was evaluated with a non-parametric test (Mann-Whitney U test) after checking for normality with the Anderson-Darling test. The statistical significance was set at 0.05.

## 4 Results

### 4.1 Sustained improvement with aDBS+ on PD-related symptoms and gait

The UPDRS-III and -IV scores were with aDBS+ 4/108 and 0/12 and with cDBS+ 11/108 and 2/12. The Gait and Falls Questionnaire score was 15/64 with aDBS+ and 34/64 with cDBS+. During a brief discontinuation (about 30 min) of DBS treatment at medication wearing-off, the patient was severely bradykinetic and unable to stand without assistance and to walk, and the UPDRS-III score was 57/108.

When reprogrammed to cDBS, the patient reported a progressive worsening of gait that started after about 3–4 days. By day ten in cDBS+, the patient reported similar worsening to the chronic cDBS treatment received before aDBS. We therefore did not continue further with the cDBS+ mode. The patient then returned to aDBS+ with recovery of gait benefit that was maintained, along with benefit on other parkinsonian symptoms, until the last follow-up at 6 months.

**FIGURE 3:**
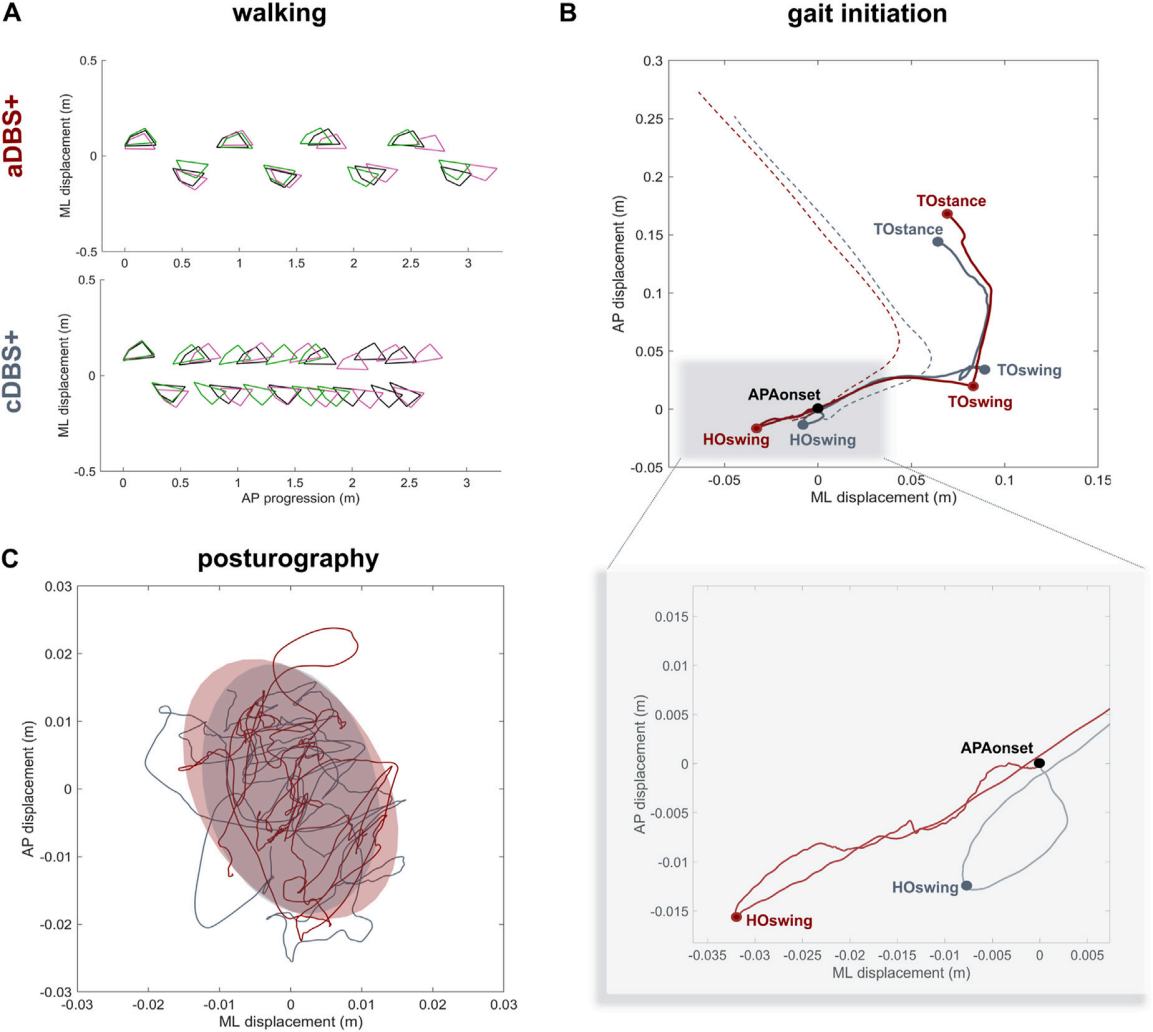
Kinematic results **(A)** Walking trials: feet placement during three subsequent trials of unperturbed walking (green, pink and black) in aDBS+ (top panel) and cDBS+ (bottom panel). To allow trial alignment, the starting anterior-posterior (AP) position of each trial was placed at the first available left heel contact, and the average value of the medio-lateral (ML) marker coordinates was removed from each track. **(B)** Gait initiation: exemplary CoP (solid line) and CoM (dashed line) pathway in the ML and AP directions during two trials of gait initiation in aDBS+ (red) and cDBS+ (grey). The two trials were aligned at the starting point of the APAs (APAonset). CoP and CoM tracks are displayed from the APAonset to the toe-off of the stance foot (TOstance).The pathway of the CoP describes the imbalance phase, from the onset of the APA to the heel-off of the swing foot (HOswing), the unloading phase, from the HOswing to the toe-off of the swing foot (TOswing), and the stepping phase, from the TOswing to the TOstance. **(C)** Posturography: CoP displacement in the ML and AP directions during 30 s of standing during aDBS+ (red) and cDBS+ (grey) stimulation. For each stimulation condition, the confidence ellipse includes 95% of the points of the CoP track. Abbreviations: a, adaptive; AP, anterior-posterior; APAs, Anticipatory Postural Adjustments; c, conventional; CoM, Centre of Mass; CoP, Centre of Pressure; DBS, deep brain stimulation; DBS+, with dopaminergic medication; HO, heel-off; ML, medio-lateral; TO, toe-off

Spatiotemporal gait parameters improved in aDBS+ condition with respect to cDBS+ (Figure 3A; Table 1). In particular, aDBS+ increased gait velocity (values are reported as mean (SD): 0.38 (0.06)m/s for cDBS+ and 0.56 (0.01)m/s for aDBS+) and ameliorated cadence (110.7 (25.11)step/min for cDBS+ and 84.76 (3.76)step/min for aDBS+), as well as significantly improved stride parameters and gait variability (Table 1). APAs at gait initiation improved with aDBS+. Specifically, we described a beneficial effect of aDBS+ on the imbalance phase, and a reduction of the unloading phase (Figure 3B; Table 1). With aDBS+, we also observed an improvement in first step length and velocity and CoM velocity at the stance toe-off (Figure 3B; Table 1). Posturography measurements did not show a clear change between cDBS+ and aDBS+, but a decreased dispersion of the movements of CoP was observed in aDBS+ (Figure 3C; Table 1).

### 4.2 STN-LFP amplitude in the patient-specific beta frequency range did not differ between cDBS+ and aDBS+

The median TEED (McAuley, 2020) in cDBS+ was 6.72·10^–8^ W for the left and 5.15·10^–8^ W for the right hemisphere and for aDBS+ was 6.23·10^–8^ W for the left and 5·10^–8^ W for the right hemisphere.

In the LFP spectra we identified a predominant spectral beta peak at 16.0 [15.6, 16.3]Hz (cDBS+) and 16.2 [16.1, 16.3]Hz (aDBS+), values are reported as median [first quartile, third quartile] (Figure 1).

The daily median and the interquartile range of the BFRA did not significantly differ between the two stimulation modalities (Median: values reported in nV as median [first quartile, third quartile], cDBS+: 321.3 [296.7, 330.1], aDBS+: 345.2 [252.9, 358.6], Mann-Whitney U test: *p* = 0.93, Figure 2E. Interquartile range: cDBS+: 171.1 [124, 199.3], aDBS+: 208.8 [169.1, 246], Mann-Whitney U test: *p* = 0.11; Figure 2F).

## 5 Discussion

Gait impairment in PD is determined by a complex interaction of pathology, age-related changes, compensatory mechanisms, and deconditioning (Wilson et al., 2019). This explains the multifaceted features of parkinsonian gait, the patient-specific electrophysiological alterations (Arnulfo et al., 2018; Canessa et al., 2020; Louie et al., 2022), and the variable and often unsatisfactory response to therapy (Curtze et al., 2015; Pozzi et al., 2022), particularly to STN-DBS, which directly modulates the activity of an essential node of the supraspinal locomotor network.

Current data about the effect of STN-cDBS on gait are heterogeneous, with only a few studies objectively assessing gait alterations (Rocchi et al., 2002; 2012; Liu et al., 2006; Nardone and Schieppati, 2006; Crenna et al., 2008; Chastan et al., 2009; Hausdorff et al., 2009; Collomb-Clerc and Welter, 2015; Cavallieri et al., 2023). Overall, STN-cDBS may improve kinematic parameters (e.g., stride length and velocity, range of motion) most dependent on levodopa-responsive symptoms, i.e., bradykinesia and rigidity. Such improvement may be expected in the first year after surgery, but gait deterioration may appear beyond 2 years after surgery (St George et al., 2010) with a worsening of gait performance already at 6 months, despite general motor improvement (van Nuenen et al., 2008).

It is still largely unknown how the STN contributes to the encoding of motor acts, especially human gait, and how this activity evolves over time with chronic DBS. Our clinical case provides the first long-term data on this topic. Notably, aDBS+ improved gait performance in an elderly subject (in his seventies) with long disease duration (>25 years) and after 10 years of cDBS+ as interesting evidence of the responsiveness of brain networks to DBS (Hartevelt et al., 2014; van Hartevelt et al., 2017).

The STN has a central role in human locomotion including fine-tuning of top-down information flow across the supraspinal locomotor network to accommodate online gait dynamics in an ecological environment. Being directly connected with the supplementary motor area (SMA) and the mesencephalic locomotor region (Nambu et al., 2002; Miocinovic et al., 2018), two main regions of the supraspinal locomotor network, the STN is expected to facilitate the necessary processing for gait adaptations.

This is particularly the case, as shown in our report, of feedforward motor programs, such as APAs, ensuring postural activities promoting gait initiation and adaptation. At gait initiation, APAs and particularly the imbalance phase, originate in the SMA (Jacobs et al., 2009; Richard et al., 2017), with a direct contribution of striatal dopaminergic tone. A previous study already showed a detrimental effect of STN-cDBS on APAs production when combined with dopaminergic drugs (Rocchi et al., 2012). In particular, cDBS may directly impact the SMA activity anterogradely (Li et al., 2007; Johnson et al., 2020), limit the cortical-subthalamic dynamic synchronization (Pozzi et al., 2019; Fischer et al., 2020), and impair feedforward motor control needed to update postural goal changes during locomotion.

We should emphasize that the aDBS algorithm used is not specific to gait, but was defined to monitor beta oscillations as a putative biomarker of PD-related bradykinetic-rigid symptoms and its fluctuations over minutes. We still know little about the evolution and changes of this biomarker, and of brain activity in general, over time with chronic cDBS or aDBS (Neumann et al., 2017). However, the preliminary observation of a similar distribution of beta power over the 10 days in the two stimulation modes (Figure 2) allows us to speculate that the benefit obtained on gait does not result from a different modulation of subthalamic beta power. In the context of human gait, subthalamic beta power would primarily code general motor acts (Koeglsperger et al., 2021; Vissani et al., 2021; Louie et al., 2022; Thenaisie et al., 2022) as part of extended neural network dynamics, such as interhemispheric coupling (Arnulfo et al., 2018) and cortical-subthalamic reactivity (Pozzi et al., 2019), frequency modulation (Canessa et al., 2020), and involvement of multiple frequencies (Koeglsperger et al., 2021). In line, a nonfixed stimulation amplitude, as applied by our aDBS mode, would be less detrimental or promote subthalamic oscillatory activity (Figure 2), and thus long-range locomotor network processing and gait restoration.

Neural oscillations reflect fluctuations of local neuronal ensembles, and their synchronization provide a mean for dynamic brain coordination (Buzsáki and Wang, 2012). This would be especially evident when online modulation of gait is required (e.g., gait initiation and stepping), whereas it would be less prominent in upright posture maintenance.

There is consensus that DBS and levodopa reduce the power of beta oscillatory activity in parallel with clinical benefit (Mathiopoulou et al., 2024). Data supporting a cause-and-effect relationship are lacking, and it cannot be ruled out that increased beta oscillations reflect a compensatory phenomenon for functional subthalamic activity in a dopamine-depleted basal ganglia circuitry. We cannot also exclude a detrimental effect of such compensatory attempt culminating in bradykinetic-rigid signs. Anyhow, it is interesting to note that beta oscillations were not abolished entirely by either cDBS or aDBS (Figure 2). Algorithms for aDBS should therefore allow for a reduction of pathological (or maladaptive) beta activity but not disrupting or flattening the physiological subthalamic beta oscillations. A linear proportional algorithm could have this advantage over “threshold” algorithms (Wilkins et al., 2024), especially in the context of complex motor tasks such as human gait. To note is that the used algorithm for aDBS does not instantaneously change the stimulation amplitude in response to detected beta power, but with adjustments based on the average of the recorded signal (see Section 3). This might suggest that it is indeed nonfixed stimulation that results in less impaired gait in chronically stimulated parkinsonian patients. However, based on our data, we cannot exclude the possibility that other nonfixed stimulation paradigms, e.g., random intermittent DBS, may prevent the worsening of gait in patients with chronic DBS. This control condition where stimulation varies in random fashion could not be applied with the current device.

New devices will allow to target more specifically the multiple network activities during human gait and gait impairment in parkinsonian patients. The quality of the recordings will be of paramount importance (Thenaisie et al., 2021). In this work, the recordings were visually inspected for the presence of artifacts, but we cannot exclude residual contamination. However, we can assume that these artifacts were similarly present in both periods with cDBS+ and aDBS+ mode, having used the same neurostimulator and having the patient conducted a similar lifestyle and daily activities.

Future studies are warranted to investigate the relationship between subthalamic oscillations, motor and non-motor PD-related symptoms, and activities of daily living by combining DBS sensing devices with wearable sensors. This would be useful not only to verify the reliability and specificity of beta oscillations over time, but also to quantify the influence of motion artifacts on recorded LFP signals.

Although this is only one patient and therefore has anecdotal value, our work opens new therapeutic perspectives for improving gait in parkinsonian patients with DBS. Identifying gait-specific biomarkers (Canessa et al., 2020) and implementing them through rapid technological development (Vissani et al., 2020) will soon result in a redefinition of neuromodulation treatments for personalized therapy at the point-of-care.

## Data Availability

The datasets presented in this article are not readily available because LFP recorded with the AlphaDBS device and kinematic data cannot be deposited in a public repository as they can be traceable to the identity of the subject. Requests to access the datasets should be directed to IUI, ioannis.isaias@asst-pini-cto.it.

## Supplementary material

Supplementary Figure S1 Evolution of the current amplitude every minute for the left (top) and right (bottom) STN during 10 days in aDBS+. For the left STN, the current was allowed to range between 3.5–4.5 mA (Amin – Amax), while for the right STN between 3–4 mA. Blue shadowed areas represent time windows of at least 30 min in which the current varied less than 2 mA (8% of the total time). System usability and technical issues caused sporadic data loss which were removed in the figure. Abbreviations: a, adaptive; A, pre-defined clinically-effective amplitude; c, conventional; DBS, deep brain stimulation, DBS+, with dopaminergic medication; STN, subthalamic nucleus.

## References

[B1] Arlotti M. Colombo M. Bonfanti A. Mandat T. Lanotte M. M. Pirola E. (2021). A new implantable closed-loop clinical neural interface: first application in Parkinson’s disease. Front. Neurosci. 15, 763235. 10.3389/fnins.2021.763235 Accessed October 25, 2023) 34949982 PMC8689059

[B2] Arlotti M. Palmisano C. Minafra B. Todisco M. Pacchetti C. Canessa A. (2019). Monitoring subthalamic oscillations for 24 hours in a freely moving Parkinson’s disease patient. Mov. Disord. 34, 757–759. 10.1002/mds.27657 30892717 PMC6593659

[B3] Arnulfo G. Pozzi N. G. Palmisano C. Leporini A. Canessa A. Brumberg J. (2018). Phase matters: a role for the subthalamic network during gait. PLOS ONE 13, e0198691. 10.1371/journal.pone.0198691 29874298 PMC5991417

[B4] Bocci T. Prenassi M. Arlotti M. Cogiamanian F. M. Borellini L. Moro E. (2021). Eight-hours conventional versus adaptive deep brain stimulation of the subthalamic nucleus in Parkinson’s disease. npj Park. Dis. 7, 88–96. 10.1038/s41531-021-00229-z PMC8478873 34584095

[B5] Buzsáki G. Wang X.-J. (2012). Mechanisms of gamma oscillations. Annu. Rev. Neurosci. 35, 203–225. 10.1146/annurev-neuro-062111-150444 22443509 PMC4049541

[B6] Canessa A. Palmisano C. Isaias I. U. Mazzoni A. (2020). Gait-related frequency modulation of beta oscillatory activity in the subthalamic nucleus of parkinsonian patients. Brain Stimul. 13, 1743–1752. 10.1016/j.brs.2020.09.006 32961337

[B7] Cavallieri F. Campanini I. Gessani A. Budriesi C. Fioravanti V. Di Rauso G. (2023). Long-term effects of bilateral subthalamic nucleus deep brain stimulation on gait disorders in Parkinson’s disease: a clinical-instrumental study. J. Neurol. 270, 4342–4353. 10.1007/s00415-023-11780-5 37208527

[B8] Chastan N. Westby G. W. M. Yelnik J. Bardinet E. Do M. C. Agid Y. (2009). Effects of nigral stimulation on locomotion and postural stability in patients with Parkinson’s disease. Brain 132, 172–184. 10.1093/brain/awn294 19001482

[B9] Chen C. C. Brücke C. Kempf F. Kupsch A. Lu C. S. Lee S. T. (2006). Deep brain stimulation of the subthalamic nucleus: a two-edged sword. Curr. Biol. 16, R952–R953. 10.1016/j.cub.2006.10.013 17113373

[B10] Collomb-Clerc A. Welter M.-L. (2015). Effects of deep brain stimulation on balance and gait in patients with Parkinson’s disease: a systematic neurophysiological review. Neurophysiol. Clin. 45, 371–388. 10.1016/j.neucli.2015.07.001 26319759

[B11] Crenna P. Carpinella I. Lopiano L. Marzegan A. Rabuffetti M. Rizzone M. (2008). Influence of basal ganglia on upper limb locomotor synergies. Evidence from deep brain stimulation and L-DOPA treatment in Parkinson’s disease. Brain 131, 3410–3420. 10.1093/brain/awn272 18952669

[B12] Curtze C. Nutt J. G. Carlson-Kuhta P. Mancini M. Horak F. B. (2015). Levodopa is a double-edged sword for balance and gait in people with Parkinson’s disease. Mov. Disord. 30, 1361–1370. 10.1002/mds.26269 26095928 PMC4755510

[B13] Değirmenci Y. (2024). Current DBS programming. Deep Brain Stimul. 4, 29–31. 10.1016/j.jdbs.2023.12.002

[B14] Donoghue T. Haller M. Peterson E. J. Varma P. Sebastian P. Gao R. (2020). Parameterizing neural power spectra into periodic and aperiodic components. Nat. Neurosci. 23, 1655–1665. 10.1038/s41593-020-00744-x 33230329 PMC8106550

[B15] Farinelli V. Palmisano C. Marchese S. M. Strano C. M. M. D’Arrigo S. Pantaleoni C. (2020). Postural control in children with cerebellar ataxia. Appl. Sci. 10, 1606. 10.3390/app10051606

[B16] Fischer P. Lipski W. J. Neumann W.-J. Turner R. S. Fries P. Brown P. (2020). Movement-related coupling of human subthalamic nucleus spikes to cortical gamma. eLife 9, e51956. 10.7554/eLife.51956 32159515 PMC7096181

[B17] Guidetti M. Marceglia S. Loh A. Harmsen I. E. Meoni S. Foffani G. (2021). Clinical perspectives of adaptive deep brain stimulation. Brain Stimul. 14, 1238–1247. 10.1016/j.brs.2021.07.063 34371211

[B18] Hartevelt T. J. van Cabral J. Deco G. Møller A. Green A. L. Aziz T. Z. (2014). Neural plasticity in human brain connectivity: the effects of long term deep brain stimulation of the subthalamic nucleus in Parkinson’s disease. PLOS ONE 9, e86496. 10.1371/journal.pone.0086496 24466120 PMC3899266

[B19] Hausdorff J. M. Gruendlinger L. Scollins L. O’Herron S. Tarsy D. (2009). Deep brain stimulation effects on gait variability in Parkinson’s disease. Mov. Disord. 24, 1688–1692. 10.1002/mds.22554 19554569

[B20] Jacobs J. V. Lou J. S. Kraakevik J. A. Horak F. B. (2009). The supplementary motor area contributes to the timing of the anticipatory postural adjustment during step initiation in participants with and without Parkinson’s disease. Neuroscience 164, 877–885. 10.1016/j.neuroscience.2009.08.002 19665521 PMC2762010

[B21] Johnson L. A. Wang J. Nebeck S. D. Zhang J. Johnson M. D. Vitek J. L. (2020). Direct activation of primary motor cortex during subthalamic but not pallidal deep brain stimulation. J. Neurosci. 40, 2166–2177. 10.1523/JNEUROSCI.2480-19.2020 32019827 PMC7055133

[B22] Jost S. T. Kaldenbach M.-A. Antonini A. Martinez-Martin P. Timmermann L. Odin P. (2023). Levodopa dose equivalency in Parkinson’s disease: updated systematic review and proposals. Mov. Disord. 38, 1236–1252. 10.1002/mds.29410 37147135

[B23] Koeglsperger T. Mehrkens J. H. Bötzel K. (2021). Bilateral double beta peaks in a PD patient with STN electrodes. Acta Neurochir. 163, 205–209. 10.1007/s00701-020-04493-5 32710183 PMC7778623

[B24] Li S. Arbuthnott G. W. Jutras M. J. Goldberg J. A. Jaeger D. (2007). Resonant antidromic cortical circuit activation as a consequence of high-frequency subthalamic deep-brain stimulation. J. Neurophysiol. 98, 3525–3537. 10.1152/jn.00808.2007 17928554

[B25] Little S. Pogosyan A. Neal S. Zavala B. Zrinzo L. Hariz M. (2013). Adaptive deep brain stimulation in advanced Parkinson disease. Ann. Neurol. 74, 449–457. 10.1002/ana.23951 23852650 PMC3886292

[B26] Liu W. McIntire K. Kim S. H. Zhang J. Dascalos S. Lyons K. E. (2006). Bilateral subthalamic stimulation improves gait initiation in patients with Parkinson’s disease. Gait Posture 23, 492–498. 10.1016/j.gaitpost.2005.06.012 16098748

[B27] Louie K. H. Gilron R. Yaroshinsky M. S. Morrison M. A. Choi J. Hemptinne C. de (2022). Decoding natural gait cycle in Parkinson’s disease from cortico-subthalamic field potentials. 2022.05.02.22274438. 10.1101/2022.05.02.22274438 PMC9663205 36270803

[B28] Mathiopoulou V. Lofredi R. Feldmann L. K. Habets J. Darcy N. Neumann W.-J. (2024). Modulation of subthalamic beta oscillations by movement, dopamine, and deep brain stimulation in Parkinson’s disease. npj Park. Dis. 10, 77–7. 10.1038/s41531-024-00693-3 PMC10997749 38580641

[B29] McAuley M. D. (2020). Incorrect calculation of total electrical energy delivered by a deep brain stimulator. Brain Stimul. Basic, Transl. Clin. Res. Neuromodulation 13, 1414–1415. 10.1016/j.brs.2020.07.020 32745654

[B30] Miocinovic S. de Hemptinne C. Chen W. Isbaine F. Willie J. T. Ostrem J. L. (2018). Cortical potentials evoked by subthalamic stimulation demonstrate a short latency hyperdirect pathway in humans. J. Neurosci. 38, 9129–9141. 10.1523/JNEUROSCI.1327-18.2018 30201770 PMC6199405

[B31] Nambu A. Tokuno H. Takada M. (2002). Functional significance of the cortico-subthalamo-pallidal “hyperdirect” pathway. Neurosci. Res. 43, 111–117. 10.1016/s0168-0102(02)00027-5 12067746

[B32] Nardone A. Schieppati M. (2006). Balance in Parkinson’s disease under static and dynamic conditions. Mov. Disord. 21, 1515–1520. 10.1002/mds.21015 16817196

[B33] Neumann W.-J. Staub-Bartelt F. Horn A. Schanda J. Schneider G.-H. Brown P. (2017). Long term correlation of subthalamic beta band activity with motor impairment in patients with Parkinson’s disease. Clin. Neurophysiol. 128, 2286–2291. 10.1016/j.clinph.2017.08.028 29031219 PMC5779610

[B34] Palmisano C. Beccaria L. Haufe S. Volkmann J. Pezzoli G. Isaias I. U. (2022a). Gait initiation impairment in patients with Parkinson’s disease and freezing of gait. Bioeng. (Basel) 9, 639. 10.3390/bioengineering9110639 PMC9687939 36354550

[B35] Palmisano C. Brandt G. Vissani M. Pozzi N. G. Canessa A. Brumberg J. (2020a). Gait initiation in Parkinson’s disease: impact of dopamine depletion and initial stance condition. Front. Bioeng. Biotechnol. 8, 137. 10.3389/fbioe.2020.00137 Accessed October 25, 2023) 32211390 PMC7068722

[B36] Palmisano C. Kullmann P. Hanafi I. Verrecchia M. Latoschik M. E. Canessa A. (2022b). A fully-immersive virtual reality setup to study gait modulation. Front. Hum. Neurosci. 16, 783452. 10.3389/fnhum.2022.783452 35399359 PMC8983870

[B37] Palmisano C. Todisco M. Marotta G. Volkmann J. Pacchetti C. Frigo C. A. (2020b). Gait initiation in progressive supranuclear palsy: brain metabolic correlates. Neuroimage Clin. 28, 102408. 10.1016/j.nicl.2020.102408 33353609 PMC7689404

[B38] Pozzi N. G. Canessa A. Palmisano C. Brumberg J. Steigerwald F. Reich M. M. (2019). Freezing of gait in Parkinson’s disease reflects a sudden derangement of locomotor network dynamics. Brain 142, 2037–2050. 10.1093/brain/awz141 31505548 PMC6598629

[B39] Pozzi N. G. Isaias I. U. (2022). “Chapter 19 - adaptive deep brain stimulation: retuning Parkinson’s disease,” in Handbook of clinical Neurology. Editors Quartarone A. Ghilardi M. F. Boller F. (Elsevier), 273–284. 10.1016/B978-0-12-819410-2.00015-1 35034741

[B40] Pozzi N. G. Palmisano C. Reich M. M. Capetian P. Pacchetti C. Volkmann J. (2022). Troubleshooting gait disturbances in Parkinson’s disease with deep brain stimulation. Front. Hum. Neurosci. 16, 806513. 10.3389/fnhum.2022.806513 Accessed April 4, 2023) 35652005 PMC9148971

[B41] Reich M. M. Brumberg J. Pozzi N. G. Marotta G. Roothans J. Åström M. (2016). Progressive gait ataxia following deep brain stimulation for essential tremor: adverse effect or lack of efficacy? Brain 139, 2948–2956. 10.1093/brain/aww223 27658421

[B42] Richard A. Van Hamme A. Drevelle X. Golmard J.-L. Meunier S. Welter M.-L. (2017). Contribution of the supplementary motor area and the cerebellum to the anticipatory postural adjustments and execution phases of human gait initiation. Neuroscience 358, 181–189. 10.1016/j.neuroscience.2017.06.047 28673716

[B43] Rocchi L. Carlson-Kuhta P. Chiari L. Burchiel K. J. Hogarth P. Horak F. B. (2012). Effects of deep brain stimulation in the subthalamic nucleus or globus pallidus internus on step initiation in Parkinson disease: laboratory investigation. J. Neurosurg. 117, 1141–1149. 10.3171/2012.8.JNS112006 23039143 PMC3990225

[B44] Rocchi L. Chiari L. Horak F. B. (2002). Effects of deep brain stimulation and levodopa on postural sway in Parkinson’s disease. J. Neurol. Neurosurg. Psychiatry 73, 267–274. 10.1136/jnnp.73.3.267 12185157 PMC1738049

[B45] Rosa M. Arlotti M. Marceglia S. Cogiamanian F. Ardolino G. Fonzo A. D. (2017). Adaptive deep brain stimulation controls levodopa-induced side effects in Parkinsonian patients: DBS Controls Levodopa-Induced Side Effects. Mov. Disord. 32, 628–629. 10.1002/mds.26953 28211585 PMC5412843

[B46] St George R. J. Nutt J. G. Burchiel K. J. Horak F. B. (2010). A meta-regression of the long-term effects of deep brain stimulation on balance and gait in PD. Neurology 75, 1292–1299. 10.1212/WNL.0b013e3181f61329 20921515 PMC3013496

[B47] Thenaisie Y. Lee K. Moerman C. Scafa S. Gálvez A. Pirondini E. (2022). Principles of gait encoding in the subthalamic nucleus of people with Parkinson’s disease. Sci. Transl. Med. 14, eabo1800. 10.1126/scitranslmed.abo1800 36070366

[B48] Thenaisie Y. Palmisano C. Canessa A. Keulen B. J. Capetian P. Jiménez M. C. (2021). Towards adaptive deep brain stimulation: clinical and technical notes on a novel commercial device for chronic brain sensing. J. Neural Eng. 18, 042002. 10.1088/1741-2552/ac1d5b 34388744

[B49] van Hartevelt T. J. Fernandes H. M. Stevner A. B. A. Deco G. Kringelbach M. L. (2017). “Chapter 25 - neural plasticity in human brain connectivity: the effects of deep brain stimulation,” in The rewiring brain. Editors van Ooyen A. Butz-Ostendorf M. (San Diego: Academic Press), 527–546. 10.1016/B978-0-12-803784-3.00025-1

[B50] van Nuenen B. F. L. Esselink R. A. J. Munneke M. Speelman J. D. van Laar T. Bloem B. R. (2008). Postoperative gait deterioration after bilateral subthalamic nucleus stimulation in Parkinson’s disease. Mov. Disord. 23, 2404–2406. 10.1002/mds.21986 18951532

[B51] Vissani M. Isaias I. U. Mazzoni A. (2020). Deep brain stimulation: a review of the open neural engineering challenges. J. Neural Eng. 17, 051002. 10.1088/1741-2552/abb581 33052884

[B52] Vissani M. Palmisano C. Volkmann J. Pezzoli G. Micera S. Isaias I. U. (2021). Impaired reach-to-grasp kinematics in parkinsonian patients relates to dopamine-dependent, subthalamic beta bursts. NPJ Park. Dis. 7, 53. 10.1038/s41531-021-00187-6 PMC8242004 34188058

[B53] Wilkins K. B. Petrucci M. N. Lambert E. F. Melbourne J. A. Gala A. S. Akella P. (2024). Beta burst-driven adaptive deep brain stimulation improves gait impairment and freezing of gait in Parkinson’s disease. medRxiv. 10.1101/2024.06.26.24309418 PMC12673813 37429355

[B54] Wilson J. Allcock L. Mc Ardle R. Taylor J.-P. Rochester L. (2019). The neural correlates of discrete gait characteristics in ageing: a structured review. Neurosci. Biobehav Rev. 100, 344–369. 10.1016/j.neubiorev.2018.12.017 30552912 PMC6565843

[B55] Yin Z. Zhu G. Zhao B. Bai Y. Jiang Y. Neumann W.-J. (2021). Local field potentials in Parkinson’s disease: a frequency-based review. Neurobiol. Dis. 155, 105372. 10.1016/j.nbd.2021.105372 33932557

