# Supplementary figures and images for: Case report: Improvement of gait with adaptive deep brain stimulation in a patient with Parkinson’s disease
Source: Front Bioeng Biotechnol. 2024 Sep 11;12:1428189. doi: 10.3389/fbioe.2024.1428189 (PMC11423205; doi:10.3389/fbioe.2024.1428189)

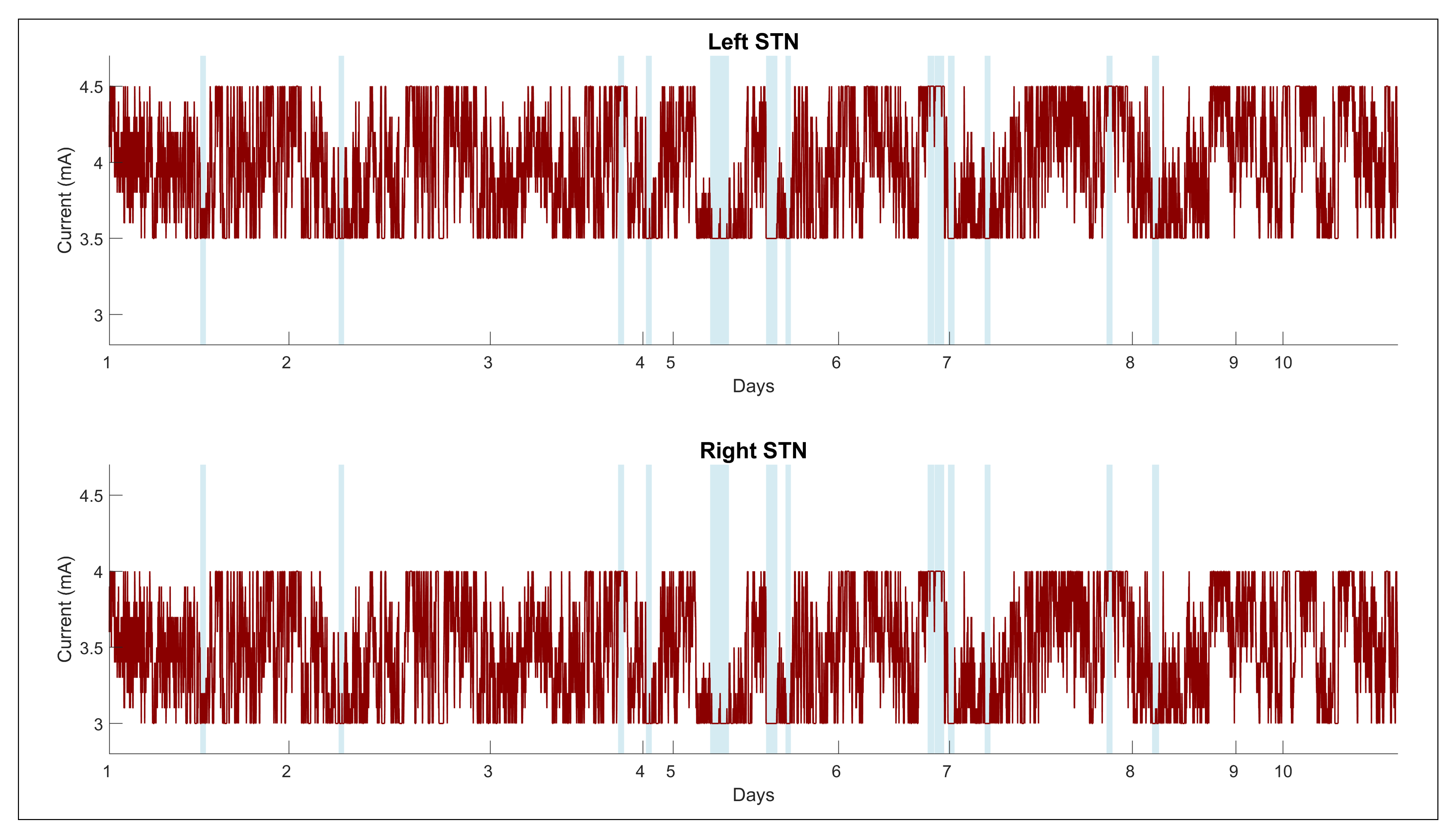

Supplement: Supplementary file 1 [file Image1.TIF]
